# “It Makes It More Real to You”: Abortion Attitudes Following Experience and Contact With Abortion

**DOI:** 10.1111/psrh.70019

**Published:** 2025-06-18

**Authors:** Julieta Baker, Nicole Lozano, Aneeka Shrestha, Ssanyu Kayser, Lora Adair

**Affiliations:** ^1^ Life Sciences Brunel University London Uxbridge UK; ^2^ Department of Psychology Angelo State University San Angelo Texas USA

**Keywords:** abortion, qualitative research methods, United Kingdom

## Abstract

**Introduction:**

When positioned as part of a cluster of related social and political attitudes, abortion attitudes are characterized as somewhat fixed from a young age. The extent to which abortion attitudes are malleable, and can be shaped by experience, is under‐researched in the United Kingdom (UK).

**Methods:**

To address this gap, we conducted semi‐structured interviews with individuals with (*N* = 12) and without (*N* = 16) abortion experience living in the United Kingdom, consisting of England, Scotland, Wales or Northern Ireland. Inductive thematic analysis was used to address the research question: How does experience and/or contact with abortion shape attitudes towards abortion?

**Results:**

The theme *From Abstract Idea to Reality* illustrates participants' understanding of how abortion attitudes are developed by contact with real, lived experiences of abortion—someone's own and/or their friends’ or acquaintances’ abortions. Participants were clear that proximity to abortion helped them, and others, to see abortion as tangible, personal, and sensory (“reality”) as opposed to intangible, imagined, and conceptual (“abstract”). Subthemes capture our participants’ understanding of abortion as a reality as opposed to something imagined; abortion is a complex issue and abortion experiences are varied (*Complexity of Abortion*), attitudes towards abortion are largely stable (*Consistency of Attitudes*), and abortion, and the people who seek abortion in the United Kingdom, is still stigmatized (*Persistent Stigma*).

**Conclusion:**

Our themes and discussion provide direction for future scholarship considering contact as a stigma reduction strategy, highlighting some potential benefits but also urging caution in oversimplifying a complicated social issue.

Abortion is a common health care service used to terminate a pregnancy, with almost 215,000 abortions carried out in England and Wales in 2021, the largest number of abortions since reporting began in 1969 [1]. Abortion has been legal in the United Kingdom (UK)—this includes England, Wales, Scotland, and Northern Ireland—since the passing of the 1967 Abortion Act, permitting abortion up until 24 weeks pregnancy gestation with approval from two independent doctors that the reason for seeking abortion care meets statutory grounds for abortion. In the absence of fetal abnormality, the pregnancy must be deemed more harmful to the health (mental or physical) of the pregnant person than if carried to term. Despite abortion occurring commonly in the United Kingdom and social attitudes becoming increasingly liberal (e.g., increasing support for gender equality in employment, education, and political representation in the United Kingdom and other high‐income countries), abortion challenges persist [2, 3]. These cluster around two main issues: barriers to abortion access and the effects of stigma at both social and structural levels [4, 5, 6, 7, 8, 9, 10]. Given the discord between the UK's legal stance and lived experiences of abortion seekers, this study was designed to explore attitudes toward abortion in the United Kingdom. Building upon recent literature that explores avenues for reducing abortion stigma and negativity, we specifically investigated how these attitudes may be influenced by contact and/or experience with abortion [11, 12, 13, 14, 15, 16].

## 1 Beyond Public Opinion—Abortion Sentiments in the United Kingdom

Since the 1967 Abortion Act, public opinion polls consistently show Britons' increasingly supportive stance toward abortion [17]. Most recently in 2022, an amendment to the 1967 Abortion Act allowed the use of telemedicine and abortion medication at home, improving access across the United Kingdom [18]. Despite the apparent liberal stance of current abortion laws, there remain barriers to abortion access: rather than permitting autonomous abortion decision‐making processes, at present the law requires pregnant individuals to receive approval from two independent doctors that their reason for seeking abortion care meets statutory grounds for abortion. Indicative of the time in which it was created, those against abortion saw it as “unnatural,” while those in favor suggested that for “desperate” women, abortion would allow them to be better mothers [19]. The Act, rather than rejecting ideas of compulsory motherhood for women, allowed exceptions where “good motherhood” was at risk [20, 21, 22]. Moreover, the innate “irrationality” of women was contrasted with the sagaciousness of (presumed male) doctors who were to become the gatekeepers of abortion after the legislation was passed [20]. This portrayal of women and pregnant people as incapable of making their own reproductive choices perpetuates a dynamic that threatens reproductive rights at various levels of British society [23]. This dynamic includes the criminalization of abortion, fostering hostile political climates, maintaining stigma, advancing the privatization of abortion care, and creating disparities in care provision [5, 24, 25, 26, 27, 28, 29]. The NHS has recently attempted to acknowledge and address the unequal distribution of abortion care and quality but, after years of disinvestment and attention, multiple stakeholders must get involved to improve abortion provision in the UK [27]. Reflecting this complexity, some data demonstrate that despite overall support for women's right to choose, many have “situationalist” attitudes, endorsing support for abortion in specific circumstances, such as “traumatic” as opposed to ‘social’ [30, 31]. Britons also show support for more restrictive legislation, including limiting abortion to 24 weeks or less [31]. This selective support is also shown in political debate and media representations of abortion, indicating that beyond a pragmatic stance on legal abortion, its acceptance and approval in society remain contested [9, 26, 32].

## 2 Why Does Abortion Stigma Persist?

According to Goffman, stigma captures the negative attributions made regarding a person perceived as violating a norm or expectation, possessing a characteristic or experience that is “incongruous with our stereotype of what a given individual should be” [33]. Abrams argues that stigma attached to these reproductive decisions [surrogacy and abortion] reflects a legacy of gendered roles and disapproval of women who fail to conform to the social expectations of motherhood [34]. Some theoretical work proposes that abortion attitudes are part of a larger cluster of beliefs about motherhood, sexuality, and femininity [35]. For example, feminist moral theory conceptualizes anti‐abortion sentiment as a consequence of pro‐natalist ideals of motherhood as natural and essential to womanhood [36]. An ethnographic study of abortion discourse in UK public spaces echoes these essential motherhood narratives, perpetuating the idea that women are meant to be mothers, and seeking an abortion is something that they are led to [37, 38]. The relationship between gendered norms and abortion stigma is bolstered by survey findings that correlate hostile and benevolent sexism, traditional gender role attitudes, and anti‐abortion attitudes [35, 39]. And, other applications of feminist theory highlight how women's identities as “good women” are challenged as their abortion experiences contradict culturally entrenched narratives that position women's sexuality as exclusively for procreation [40], providing a valuable framework to conceptualize abortion as a stigmatized experience, given that abortion challenges the norms and expectations of womanhood [41].

Stigma around abortion discourages people from sharing their experiences, particularly within their circle of family and friends, exacerbating the idea that abortion is an exceptional experience [42, 43, 44], isolating people from engaging with a support network and perpetuating abortion myths. Indeed, a survey study finds that those who do not know anyone who has had an abortion are more likely to lack accurate abortion knowledge [45]. Beyond facilitating feelings of shame and secrecy around one's abortion experience, abortion stigma has negative consequences on mental health, such as increased stress, anxiety, and depression [46, 47]. Additionally, if abortion stigma is felt during interactions with healthcare professionals, it can act as a barrier to accessing needed reproductive healthcare services [6, 48]. Gendered norms and expectations likely preserve less supportive abortion attitudes even in a context where abortion is legal and relatively accessible.

## 3 Can We Change?

Cross‐culturally, abortion attitudes have been linked to broader beliefs about morality, sexuality, and social/political views [49]. Applications of political theory conceptualize abortion attitudes as an outcome of “symbolic predispositions,” such as social/political conservatism, conformity to traditional gender roles, and religiosity. Symbolic predispositions, or “symbolic politics,” refer to how individuals construct their attitudes and preferences about various issues [50]. This perspective proposes that individuals are socialized into particular groups and ideologies (e.g., political party affiliation, liberal or conservative political ideologies, religious affiliations, and beliefs) from a young age and use this predisposition as a heuristic tool to construct attitudes and beliefs about issues confronted in the future. Indeed, in the United Kingdom, negative media portrayals of abortion and narratives from anti‐abortion movements frame abortion as a religious or moral issue and position morality as intrinsic to the abortion debate [26, 37]. Consistent with a symbolic politics model, research finds that abortion attitudes, measured as support for abortion in various contexts and stance on various pro‐/anti‐abortion arguments, are predicted by various indicators of symbolic predisposition, including political party affiliation, religious fundamentalism, and sexual liberalism (e.g., attitudes toward LGBT+ people, individuals' approach/avoidance of sex‐relevant topics and issues) [39, 51]. This symbolic political approach to understanding abortion attitudes implies that they are somewhat fixed.

Stigmatized attitudes towards many experiences and characteristics, such as same‐sex/gender sexuality, ethnic minority group membership, refugee status, and transgender identity, can be attenuated by contact (e.g., friend, acquaintance, work relationships, electronic contact via chat rooms and forums) with individuals with those experiences and characteristics [52, 53, 54, 55, 56, 57, 58]. This strategy has been tentatively explored in the context of anti‐abortion sentiment, which finds positive impacts of group contact on stigma [16, 59]. This phenomenon is explained by intergroup contact theory, specifically the contact hypothesis, which posits that stigma between individuals belonging to different social groups can be reduced if there is contact between the different groups [60, 61]. According to foundational research by Allport, these encounters can be even more efficacious at reducing stigma if they are characterized by cooperation, equality, and shared objectives; some meta‐analytic findings suggest that these conditions do not have to be met for contact, even imagined or vicarious contact, to reduce stigma [57, 60, 62]. While intergroup contact can reduce stigma, the research has faced significant criticism, highlighting the need for continued scrutiny. Critics point to issues such as the challenge of replicating positive effects at scale, prevalence of publication bias, omission of key variables, and difficulties in implementing contact interventions in unstructured, everyday contexts [62, 63, 64].

The mechanisms driving the connection between contact with individuals from different social groups and reduced stigma are complex and varied. Contact has been the least investigated method for reducing abortion stigma, and due to the deeply contextual nature of abortion attitudes and experiences, much more research is needed if contact is to be considered a stigma reduction strategy in the United Kingdom [65]. It is clear that abortion stigma persists in the United Kingdom, but with little literature exploring abortion attitudes in the United Kingdom and methods for stigma reduction, the question remains: *Can abortion attitudes be changed?* We sought to explore this by looking at how contact with lived experiences of abortion, either through a personal abortion or a friend or acquaintance's abortion, influenced abortion attitudes and beliefs.

## 4 Purpose of the Study

This study used a reflexive, feminist approach to explore the research question: How does experience and/or contact with abortion shape attitudes towards abortion? Through a semi‐structured interview study with women in the United Kingdom, we sought to access our participants' understanding of how contact with lived experiences of abortion, whether a person's own abortion or that of someone they know, might shape their understanding of abortion and/or people who receive abortion care, willingness to accept or offer social support, and empathic concern for those who receive abortion care.

## 5 Methods

The findings reported here are part of a larger study, which applied semi‐structured interview methods to explore abortion decision‐making processes and experiences of UK women, whether theirs or others. Abortion attitudes/judgments were probed throughout the process. Results that relate to participants' abortion judgments, specifically how acceptable they think it is to access abortion under various circumstances when considering both themselves (what is acceptable for me) and others (what I find acceptable for someone else), are reported by Lozano et al. [31]. In this paper, we detail findings that have not been reported elsewhere, related to our participants' understanding of the malleability of abortion attitudes, following experience and/or contact with abortion.

### 5.1 Participant Selection

We conducted interviews from September 2021 to May 2022 with 29 participants. We employed multiple recruitment strategies, including advertisements in reproductive health clinics, social media postings, and snowball sampling. To participate, a person needed to be able to speak English, be over the age of 18, and have had the ability to be pregnant during their lifetime. Specifically, we aimed to recruit participants where abortion would be a relevant form of healthcare (i.e., they have been capable of experiencing pregnancy at some point during their lifetime) and to sample those with and without personal abortion experience.

### 5.2 Tool Development

We developed two interview protocols based on the research questions as well as extant literature exploring abortion decision‐making [42, 46, 66]. Specifically, protocols were designed to address three related research goals: (1) to gain an appreciation of the nuance and complexity of abortion attitudes, (2) to understand abortion decision‐making processes, and (3) to explore social aspects of abortion (e.g., social support, stigma). One protocol was designed for participants who had at least one abortion, and one protocol was designed for participants without an abortion experience. Examples of questions include, “Do you think having an abortion changed your beliefs or expectations about abortion?,” “Do you think it may have changed the way you think and feel about abortion?,” “Do you think that knowing someone who has had an abortion changed any of your views or your understanding of abortion?” We utilized a semi‐structured interview format to allow flexibility in conversations with participants [67]. Though the guides were not piloted with participants, the research team discussed the first interviews to assess if the guides were providing rich data. Additional details, including the full interview guides, are reported in Lozano et al. [31].

### 5.3 Data Collection

Participants completed a short demographic survey before scheduling an interview, with all interviews held via Zoom or telephone based on individual preference. Though we completed 29 interviews, we removed one interview from our analysis due to recording issues, leaving 28 participants in the sample. Interviews averaged 40 min in length; two members of the research team transcribed interview recordings and replaced participants' names with pseudonyms from an online baby name generator; the entire team coded these transcriptions.

### 5.4 Data Analytic Strategies

Data analysis followed Braun and Clarke's [68] recommendations for reflexive thematic analysis (TA). Reflexive TA emphasizes the active role of the researcher in the coding and theme development process, as well as recognizing the subjectivity that is brought to the qualitative process. This method ensures that the data can be coded in a way that iteratively accounts for participants’ perspectives and the research question (How does experience and/or contact with someone's abortion shape attitudes towards abortion?), and asks the researchers to understand how their personal, functional, and disciplinary reflexivity participates in the knowledge production. The general analytic process involves six stages: (1) dataset familiarization, (2) data coding, (3) initial theme generation, (4) theme development and review, (5) then refining, defining, and naming, and (6) writing.

Five researchers engaged with the coding process, relying on semantic coding as well as latent coding [46]. The interviews were coded utilizing an inductive approach—meaning that the team did not analyze the transcripts with a specific theory in mind but rather allowed the findings to develop as we worked through the transcripts. Initially, over 3500 codes were developed from the interviews following Braun and Clarke's process [46]. As themes were generated and refined, we focused on capturing participants’ unique points of view, actions, meaning (both what was directly said and what was implied) and understanding—this included interpreting participants’ underlying meaning, beyond semantic codes, to capture deeper conceptual or latent content in interview transcripts.

### 5.5 Methodological Integrity

The authors used elements of Guba and Lincoln to establish trustworthiness [69, 70]. Trustworthiness was established through several mechanisms, including engagement (i.e., building connection and trust with our participants), reflexivity (i.e., addressing researchers' understanding, values, and assumptions throughout the research process), an audit trail (i.e., recording each step of the data collection and analysis process), and a commitment to producing thick descriptions. While we reached data saturation in our sample, we do not claim generalizability of the findings. To aid the transfer of these findings, detailed or ‘thick’ descriptions of participants' understanding of the relationship between abortion experiences (their own or someone else's) and abortion attitudes are included. We conceptualize data saturation [71] as finding no new information in the transcripts (e.g., the meaning of specific codes remained stable upon successive interviews) and understanding participants’ experiences and perspectives.

### 5.6 Reflexivity

Following Reflexive TA, reflexivity was a continual part of the research process. The team consisted of two PhD researchers, who have expertise in reproductive decision‐making and gender‐related issues. Several students assisted with the project, including three of the authors, each having various research interests including reproductive decision‐making, the role of social support (or the lack thereof) in shaping reproductive and sexual experiences, and evolutionary social sciences. Prior to the data collection process, the team discussed their views, experiences, and values regarding abortion. This included discussing how those values influence the interview process (e.g., what questions we would decide to follow‐up on, as well as how we decided what questions on which to focus) and again how it could influence the coding process. We discussed how we were raised (such as two members of the team being raised in the US South; other members having strict rules regarding sex education) and which value systems regarding pregnancy, sex, and parenting we have been able to develop for ourselves. As we identified and developed themes, the research team would identify reactions we were having and how the various experiences we had through interviews with participants and through our work were impacting our interpretations.

### 5.7 Findings

Using semi‐structured interviews, we asked participants how their own and others' experiences of abortion might shape their understanding, thoughts, and feelings about abortion. Though trans and non‐binary participants were not excluded from recruitment, the sample consisted of only cisgender women ranging from 18 to 73 years old. Most participants identified as White/British and were in relationships (see Table 1 for full demographics). Twelve participants reported having had an abortion previously. Through reflexive TA, using an inductive approach, we construed several themes from our participants' responses. Here, we focus on the theme *From Abstract Idea to Reality* and its sub‐themes: *Complexity of Abortion, Persistent Stigma*, and *Consistency of Attitudes*. These themes encapsulate how our participants described the development of their understanding of abortion through lived experiences. Initial perceptions of abortion, once abstract and simplified, became more complex and tangible, acknowledging stigma and reaffirming supportive abortion attitudes through lived experience.

**TABLE 1:** Participant demographics.

Pseudonym	Age	Abortion experience	Number of children	Relationship status
Riley	29	Personal and relational	0	In a relationship (dating)
Hazel	25	Personal	1	In a relationship (casually dating)
Madison	40	Personal	2	In a relationship (married)
Willow	45	Personal and relational	2	Casually dating
Ivy	73	Personal and relational	3	Single (widowed)
Audrey	39	Personal	0	In a relationship (dating)
Hayden	39	Personal and relational	0	In a relationship (married)
Emery	50	Personal and relational	1	In a relationship (married)
Ashton	47	Personal	3	In a relationship (married)
Everleigh	32	Personal and relational	3	In a relationship (married)
Ember	26	Personal	0	Casually dating
Wren	32	Personal	0	In a relationship (married)
Ashley	22	Relational	0	Casually dating
Evelyn	28	Relational	0	Casually dating
Harper	25	Relational	0	In a relationship (married)
Avery	45	Relational	3	In a relationship (married)
River	54	Relational	2	In a relationship (married)
Easton	27	Relational	0	Single (not seeking a relationship)
Everly	43	Relational	2	In a relationship (married)
Kinsley	27	Relational	0	In a relationship (married)
Blake	48	None	3	In a relationship (married)
Hailey	42	Relational	2	In a relationship (married)
Jade	27	Relational	0	In a relationship (dating)
Oakley	46	Relational	0	Single (seeking a relationship)
Hadley	65	Relational	0	Single
Piper	20	Relational	0	In a relationship (dating multiple people)
Daisy	18	Relational	0	In a relationship (dating)
Summer	41	Relational	0	In a relationship (dating)

### 5.8 From Abstract Idea to Reality

The theme, *From Abstract Idea to Reality*, reflects how contact with abortion can affect a person's attitude toward abortion by making this health care service feel more “real” (tangible, personal, and sensory) and less abstract (intangible, imagined, and conceptual). Our participants’ narratives highlight how contact with abortion took them from a conceptual or theoretical space such as a tabloid heading or as part of political discourse into a multifaceted reality you can see, touch, and feel. Abortion as a reality, to our participants, had the attributes of a thing that can be experienced or observed (complex, has personal relevance, nuanced, etc.), as opposed to something imagined or contrived. Even so, participants acknowledge that stigma persists with regard to abortion, and that contact with a proximate abortion story (a friend, co‐worker, and/or acquaintance's abortion story) or experiencing abortion for themselves affirmed their previously held attitudes.

Participants shared how their own or someone else's abortion experience had impacted their abortion attitudes. According to our participants, abortion is not something that can be fully understood without a personal connection:

If you haven't personally been through it, then you wouldn't really know exactly what it's like. [Harper]

…until you've actually felt something yourself or been in that situation… it's difficult to understand. [Riley]

When asked about the potential impact of personal experience and/or contact with proximate abortion stories, Kinsley explains that people transition from a simplified “pro‐choice” positionality, which she attributes to childhood experiences, to a more complex view: “from my more liberal upbringing and seeing people go through it… there's a lot more reality to the situation… it's not just a theoretical thing.”

When asked how knowing someone who has had an abortion might change opinions about abortion, Jade told us that:

having that knowledge and connection with someone would make it easier to make the decision for an abortion… it helped give you the strength to know in your own life; [that] there are people around you who have had to face this decision as well, so it's not… an abstraction.

When asked the same question, Easton similarly highlighted that “… when things go from an abstract moral panic to ‘this is someone that I know, and these are the reasons that they did what they did’ … it makes it more real to you and more personal.” Our three subthemes (*Complexity of Abortion*, *Persistent Stigma*, and *Consistency of Attitudes*) delve deeper into our participants' understanding of abortion as a reality as opposed to something imagined: seeing abortion as diverse, colored by stigma, and reaffirming their stance toward structural support.

### 5.9 Complexity of Abortion

The sub‐theme *Complexity of Abortion* reflects the diverse range of experiences surrounding abortion, encompassing a range of reasons, processes, and outcomes. This aspect of abortion reality (i.e., that reality is complex while abstract ideas are simple) is a key feature in our participants' descriptions, specifically capturing how prior to experience, their understanding of abortion was narrow, theoretical, and often simplified the abortion process, their understanding of abortion as a moral issue, and their understanding of the people who seek and engage with abortion care. Participants' perception of abortion before these illuminating experiences was often described as a simplistic, often binary, political conceptualization:

I identify as a leftist person, and I'm not religious to …So, I have always felt that abortion is a human right [Hayden] and

It's more about learning about women's rights. I'm a feminist, so I really do believe in equal rights … It kind of comes from that. [Harper]

After experiencing abortion themselves or through contact with someone else's abortion, their understanding expanded to include variety in abortion experiences, including appreciating the variety of emotions people may experience, unique needs, wants, and circumstances that might bring them to seek abortion care, and the practical realities of engaging with abortion care. As an example, Everleigh explains that “prior to having a termination myself, I think I was one of those people that always thought I wouldn't do it,” describing a persistent sense that abortion was not an option for her: “I went to a Catholic school… my grandparents were quite religious… I think you already have that installed in you… having those opinions put on you… you always think… if I was in that situation I don't think I could do it.” Contact with abortion through her career expanded her understanding of abortion, and the conditions and circumstances that bring people to seek out abortion care: “… working in that area now as well I've got a bigger understanding of the background and reasonings behind why people do it. I say I am… really [am] advocate for it.” Oakley similarly shares an expanding understanding of abortion through contact with abortions in her professional/outreach work: “I respect any woman because we do not know their stories…I work in [healthcare]…for me, since I've been here, you hear stories, what people goes through…in the job we do, you cannot judge people.”

Hadley describes this deepening of her understanding metaphorically: “I think I was probably more black‐and‐white about it [abortion] before. And now I can see the shades of grey and the many shades of grey.” Here, she describes how her understanding of abortion changed to recognize its complexity after her flatmate had an abortion. Emery also describes this deepening complexity, though maintaining her supportive stance, starts to feel more discomfort and uncertainty after her own experience of pregnancy, (referencing her “maternal instinct and feeling a bit more attached [to the foetus]”), questioning whether she would choose abortion in different circumstances: “abortion, it was part of this kind of very obvious decision and the way that I was brought up [very liberal, pro‐choice]…I started to feel… not happy with that, you know just because [an unplanned pregnancy] is not ideal and just because abortion is an option it doesn't mean that I… should stop it. So, a little bit more … if you want, moral and unclear.”

Several participants noted that contact with abortion broadened their understanding of medical processes, the physical experience for the pregnant person, and the pregnant person's emotional support needs. Many had initially misunderstood the abortion process, often assuming more flexibility (e.g., less gestational restrictions), rapidity, and ease, only later learning the complexities involved. Kinsley explains that

… when I was younger, it was just like, oh you just… take a pill at any point in pregnancy and… it just… stops” and now she knows “… a little bit more about the actual medical side of how the abortion works…it's not just a… quick thing.

When reflecting on her own abortion, Hazel shares:

What I learnt actually from that, and the whole experience is that someone really needs support, constant support, emotional, especially emotional and mental someone will require support.

Hayden's abortion experience deepened her understanding of abortion processes as well, transitioning her from a state she describes as initially “immature… I didn't even think about… properly what abortion meant” to her second abortion experience wherein she was “much more confident.” This theme depicts how our participants’ contact with abortion experiences broadened their understanding of abortion in appreciating its diversity.

### 5.10 Persistent Stigma

Although recent policy changes in the United Kingdom have increased abortion accessibility, and the UK public is generally considered to have increasingly supportive abortion attitudes, our participants highlighted that abortion stigma persists. Participants were aware that those who have accessed abortion care are likely to face judgment through close and more distant social connections. Many had been a target of or witnessed abortion stigma and reported using secrecy and strategic disclosure to manage it. It was clear that stigma was a key feature of participants' understanding of abortion as a reality, rather than something conceptual or imagined. To illustrate, Piper refers to this paradox of personal experiences of stigma and more general discourse about the United Kingdom becoming increasingly progressive and accepting when she explains that:

…even though we're such a progressive generation, people are still judging people for their decisions that has nothing to do with them.

Some of our participants mentioned the existence of stigma in relation to moral and ethical ideals. When reflecting on the challenges with her own abortion experience, Everleigh shares that:

… there's this big stigma around [abortion]… I think if it wasn't seen as this big ethical dilemma then people would feel more at ease making the decision and then feel better about it afterwards.

Similarly, Ashley contextualizes anti‐abortion sentiment as a consequence of a lack of experience and specific ethical ideals: “… people that have not had an abortion… they'll view you in a different way. Definitely people will judge you for what you did, especially if they believe that abortion is kind of a sin or something wrong to do.” Others spoke of stigma more broadly: when describing how in control of her reproductive future she feels, Harper shares that she thinks she “would be judged” for seeking abortion care, that “co‐workers… friends maybe… they probably wouldn't say it out loud but… they'd… be thinking… ‘oh my god, she's had that done. That's not right.’” There was a concern about having an abortion for the “right reasons” though most participants struggled to identify the right reason.

Many participants mentioned experiencing or anticipating judgment from family, peers, and communities; “I'm sure they [friends] absolutely know, but I have never told them, purely because you just don't know what other people's thoughts are and you don't want to be judged.” [Madison], “I wouldn't really go to any place that is near my area. I just have to go somewhere far away, where people don't know me, and not there to judge me” [Ashley], and “there's a lot of stigmas around mental health illnesses and disorders…like abortion, and these things aren't necessarily discussed, like actively in families and openly in like communities” [Riley]. Some avoided, or anticipated avoiding (for a future abortion), disclosing their abortion experiences to avoid stigma, with Oakley stating: “I think I'll just go quietly and do it and just be quiet and not telling anybody.” Others witnessed stigma in educational settings, such as Piper, who recalled a classmate being taken out of school due to judgment after an abortion: “she took a lot of time off school as well… it was just… so taboo… people will use it [in] arguments… ‘at least I didn't kill a baby’ and stuff like that.” Participants understood that abortion stigma was contextual, with some abortion decisions more likely to be moralized than others. To illustrate Kinsley and Riley explain that, respectively: “[abortion would be met with] slightly more judgment if it was kind of done when someone was being careless with, you know, their birth control or not really planning for those kinds of things and in a pretty stable relationship”; “[at school] there was like a whole stigma around that girl that had three abortions and that she's just a fucking idiot.” All participants recognized that abortion still carried stigma, which, for some, influenced how they navigated their abortion experience. Our final theme examines how contact with abortion shapes participants’ attitudes toward it.

### 5.11 Consistency of Attitudes

This sub‐theme reflects participants' belief that personal abortion experience affirmed or strengthened previously held opinions about abortion. While abortion experiences were diverse, participants were clear that their views—whether supportive or opposed—remained consistent before and after their own abortion experiences. Some perceived that this confirmation was not an attitude or opinion “change,” while others reflected that they felt a shift as their opinions and convictions were strengthened. It was clear that while participants understood that experiencing abortion themselves and/or contact with abortion influenced their way of thinking about abortion, making it more “real” (tangible, personal, complex practically, and morally), they also felt that their own attitudes were largely consistent.

When asked if having an abortion changed her beliefs or feelings about abortion, Hazel reaffirms her position as opposed to abortion in cases where the health of the pregnant person and/or fetus is not at risk:

I don't think I've changed… because my reason for abortion was a critical [health condition] and that is what I believe already… that whoever wants to have an abortion should be in a critical condition, that an abortion is the only option.

When asked the same question, Emery, Ivy, and Ashton are adamant that their attitudes have not changed. To illustrate, Ivy says “no… I've come alongside people who've [needed abortion care] and I've been able to share with them… come alongside and support and… allow them to have somebody to talk to.” Ivy describes her attitudes as firmly “pro‐choice”: “I think women should have a choice as to what they do with their body” and continues to emphasize the importance of decisional autonomy after her own abortion, “I've been very careful not to inflict my opinions on them [friends considering abortion].”

Some of our other participants were also explicit about the role that their own abortion experience played in strengthening their original attitudes. When reflecting on her abortion, Wren shares that “it [made] my feelings around abortion stronger… that should be decided by the person that ends up getting pregnant… because it's their health and it's their right to make that decision for themselves.” Everleigh's original “pro‐choice” stance was strengthened, and she explains feeling “even more open” after her own abortion experience. Similarly, reflecting on her own abortion, Madison shares that she's “always believed it's the woman's choice…” and “having now done it myself… I would make my feelings known [to women considering abortion care] that this is your decision, and I am supporting you in that decision.” This final sub‐theme encapsulates how contact with abortion experiences reinforced our participants’ pre‐existing beliefs, whether pro‐choice or opposed. This consistency in attitudes highlights the complexity between personal experience and belief systems. Within the context of stigma reduction strategies and broader societal implications we explore this dynamic in the following discussion.

## 6 Discussion

Through Reflexive TA, we identified key themes that describe our participants' experiences. Our overarching theme, *From Abstract Idea to Reality*, encapsulates how participants' understanding of abortion evolved after contact—either through their own experiences or by observing the abortion experience of someone else. Before this contact, abortion was often perceived as a theoretical, politicized, oversimplified, and stereotyped issue, shaped by assumptions that framed it as more acceptable primarily to young, irresponsible, and/or sexually unrestricted individuals. Participants reported having little comprehension of the diverse and complex realities (e.g., tangible, personally relevant) of abortion, including what abortion is like (practically, physically, emotionally, etc.), who seeks it, under what circumstances, and within what contexts. After contact, however, their perspectives became more grounded in these experienced realities, and they were therefore able to recognize abortion as a complex and multifaceted experience with diverse outcomes and processes.

Our subthemes further delineate this evolving understanding. *Complexity of Abortion* highlights how first‐hand experiences enriched their perspectives, shedding light on the diversity of abortion experiences and its societal implications. In our discussion, we situate these findings within the broader literature on abortion‐related challenges and potential strategies for destigmatization. *Persistent Stigma* explores how participants encountered or observed stigma, even in an environment where abortion remains legal and accessible. *Consistency of Attitudes* examines how participants reaffirmed their stance on abortion as they integrated personal insights and contextual knowledge.

Our subtheme *Persistent Stigma* highlights the persistent nature of abortion stigma, despite recent policy changes in the UK that have expanded access through the distribution of mifepristone and misoprostol for at‐home use. This enduring stigma persists even as the United Kingdom is often conceptualized as a supportive environment for abortion; indeed, data indicate that a growing majority of the population supports abortion [25]. Abortion practice occurs commonly in the United Kingdom [72, 73] but many participants described how stigma is still a key feature of abortion realities in this context. This stigma was contextually sensitive, with some abortion decisions described as more moralized than others. This aligns with research that has consistently shown people support abortion more to protect the pregnant person's life than for other reasons (e.g., the person doesn't want to continue the pregnancy or financial challenges) [7, 74, 75]. This pattern reflects the historical underpinnings of UK abortion law, which has traditionally framed abortion as an unnatural act sought by desperate or irrational women, and therefore only acceptable when sought for physical and not social, psychological, or other personal reasons [19]. Such moralization, deeply embedded in societal narratives, was evident in many participants’ accounts, reinforcing findings from prior research on the stigmatizing effects of abortion discourse [76, 77].

Participants described a range of stigma‐related consequences, spanning both individual and community levels. On a personal level, they reported experiences of overt name‐calling, ostracisation, and social isolation. At the community level, stigma contributed to widespread misinformation, limiting education and awareness about abortion and access to services. Many participants noted that they only gained an accurate understanding of abortion processes and outcomes after witnessing others' experiences or having an abortion themselves. Existing literature has well‐documented the silencing effect of abortion stigma, which discourages open discussion, restricts access to information, and reduces social support [43, 44, 76, 78]. It is clear from our participants that abortion stigma prevails even in a legally permissive environment. While much attention has rightly been on policy and healthcare provision, and we recognize that an integrated approach to reproductive rights is needed at every level (individual, community, organizational, and structural). Our participants' narratives illustrate a particular need for continued attention on how to tackle abortion stigma from a cultural and psychological perspective [5, 21, 24, 25, 79].

Among the backdrop of abortion stigma remaining in the United Kingdom and efforts to consider stigma reduction strategies, interestingly, our second theme *Consistency of Attitudes* illustrates how our participants' attitudes towards abortion were reinforced by personal contact with abortion [80, 81, 82]. Although our analysis was inductive, with meaning derived from a bottom‐up approach, this study was developed with recent literature drawing upon contact theory as a potential benefit to reducing abortion stigma in mind [16, 59, 83, 84]. This finding presents an interesting and divergent outcome to some pre‐existing literature on contact theory and abortion stigma. In efforts to explore abortion stigma reduction strategies some literature has supported the use of contact theory; that contact between individuals belonging to different social groups can reduce stigma [60, 61]. In our study, participants' experiences support claims from contact theory about the complexity of group dynamics and that both positive and negative effects of interactions (e.g., compassion through understanding different circumstances, spreading of misinformation) can coexist [6, 64]. Contact with abortion experiences generally led to a deeper and more complex and grounded understanding for our participants; however, differently to the existing literature, the effects were not universally positive. Some participants reinforced supportive views, while others encountered or perpetuated stigma. Our findings suggest that contact alone is insufficient for reducing abortion stigma. This supports critics of contact theory that highlight key issues with reproducibility and controlling the study environment enough to truly elucidate whether contact can be considered a stigma reduction strategy at all [85, 86]. We support calls for further research into other stigma reduction strategies and urge caution before definitively endorsing group contact as a method for stigma reduction. Structured interventions—such as comprehensive education and media representation—may be more appropriate methods to shift attitudes more broadly.

The consistency of abortion attitudes can perhaps be explained instead by attitude change and persuasion literature, which overwhelmingly finds that attitudes are resistant to change (due to various processing biases, such as confirmation bias [87, 88]) and that individuals' opinions and expectations can shape their interpretation of new experiences (including abortion experiences [89]) in ways that keep new experiences in line with prior beliefs. The attitudinal consistency of our participants is consistent with literature showing that abortion attitudes are particularly resistant to change. Links between abortion and broader belief systems such as religion have been investigated in the United Kingdom, where people seem to legally accept abortion despite their religious position [74, 90, 91]. Studies propose that less supportive attitudes toward abortion are persistent due to their entrenchment into religious ideologies [92, 93]. A recent study found that religious beliefs, specifically believing in a moralizing God(s) that rewards good people and punishes bad people, were associated with less supportive abortion attitudes in the United Kingdom [31]. Research in the United Kingdom has shown that across generations, religion is a key framework by which less supportive abortion attitudes are maintained [94, 95]. More recently, anti‐abortion attitudes have been shown to be particularly associated with conservative political affiliation also [96]. However, the relationship between abortion attitudes and political affiliation or orientation in the United Kingdom is unique when compared to other Western contexts. For example, while literature in the US explicitly makes these connections, in the United Kingdom, less supportive attitudes towards abortion have not been linked to traditional and sexist gender attitudes, which are often associated with a more right‐wing political stance [21, 38, 96]. Taken together, abortion attitudes are embedded among other networks of attitudes and ideas. Various mechanisms—including religious ideologies and politics—play a role in supporting attitudinal consistency before and after personal experience with abortion.

Considering the challenges presented by contemporary literature and the findings of this study, we must continue to explore potential solutions for destigmatizing abortion and, with it, improving access to accurate abortion education and support [97]. Notably, a recent campaign was launched to decriminalize abortion; although rejected by parliament, this campaign was overtly supported by recognized institutions such as the Royal College of Gynecology and Obstetrics. Its rejection from parliament, in addition to our participants narratives, indicates an additional need for normalization techniques targeted at individual and community levels [98, 99].

Within our theme *Complexity of Abortion* many of our participants drew distinctions between abstract political discourse around abortion (which they saw as oversimplified, binary, or vague), and the “reality” of abortion (which they saw as complex, diverse, and detailed) that you appreciate when you experience abortion or have contact with a proximate abortion story. As shown in the results these experiences add detail and new dimensions to our participants' understanding; people require abortions for a range of reasons and experience a variety of emotions afterward. The experiences of our participants reflect existing literature on the diversity of abortion experiences and beliefs: for example, the multitude of emotions that can be experienced towards abortion at any one time and the nuance of attitudes, that those on either side of the spectrum often hold conflicting beliefs at the same time [68, 74, 82, 83]. Our participants' understanding is that abortion stories, especially those of someone in their social circle, make abortions more real (i.e., tangible and personal), and thus a less “othered” or exceptional experience. It highlights that abortions are not medical interventions that happen to other people; they are an ordinary and common form of healthcare for people from all backgrounds, in all kinds of circumstances [100]. Highlighting abortion as a normative and acceptable experience is a key future angle that destigmatization strategies must consider.

Exploring methods of abortion normalization, the literature has primarily focused on educating both medical professionals and patients about abortion care [101, 102]. Expanding on the recognized benefits of adult abortion education, Bloomer, O'Dowd, and McLeod explored how a Foucauldian feminist approach to normalization can challenge and shift abortion stigma while allowing individuals to maintain their religious beliefs within a Northern Irish context [103]. Although still in its early stages, their findings suggest that, particularly where unsupportive abortion attitudes are held in connection to religious beliefs and ideologies, further research and funding could explore this framework as a potential strategy for normalizing and destigmatizing abortion at both individual and micro‐community levels. Purcell and colleagues employed a qualitative methodology to examine non‐negative language surrounding abortion and potential normalization strategies [14]. Their findings align with ours, indicating that negative and positive abortion narratives often coexist. However, they suggest that normalization could be achieved by defaulting to positive abortion language in media depictions to broaden cultural narratives available—allowing individuals to describe their abortion experiences using ambivalent and positive language. While the findings of our study do not support contact theory, contact with proximate abortion stories may still serve as a pathway to expand cultural narratives and language around abortion. Overall, our findings align with those of others, highlighting the need for further research to support the normalization of abortion as a destigmatization strategy [103, 104, 105].

## 7 Limitations

We aimed to uphold a high standard of trustworthiness and credibility throughout our research process, but inevitably there are some limitations. First, our data is from a sample of 28 participants, collected exclusively in the United Kingdom. As a qualitative study, it is not meant to be generalizable, but rather to explore the shared experiences of this sample. Second, abortion is legal and relatively easy to access within the United Kingdom [22, 106, 107]. This was mentioned by several participants who expressed their appreciation for the ease of obtaining an abortion legally and safely. These institutional, cultural, and legislative differences will change the way people experience abortion care and thus change the way they engage with our research question [108, 109]. Some participants were recruited via snowball sampling, which is a useful strategy—particularly when conducting research on topics and experiences that are perceived as stigmatized, sensitive, and/or personal [110]—yet likely reduced the diversity of our sample regarding study‐relevant characteristics. For example, given the researchers' involvement with reproductive health organizations and charities, it is likely that our sample contains an overrepresentation of individuals with supportive attitudes. Due to the homogenous nature of our sample and our priority to focus on rich descriptions of individuals, we have refrained from making comparisons between our data set and the general United Kingdom population. However, the homogeneity of our sample may be framed as a strength, rather than a weakness, given that it likely assisted with our ability to reach data/meaning saturation—concluding that we have “heard it all,” constructing stable codes, and ultimately producing rich descriptions of our participants' perspectives and understanding [111].

## 8 Implications and Conclusion

This study used qualitative research methods to explore how abortion attitudes are impacted by abortion experience and/or contact with abortion, among women in the United Kingdom. Following inductive thematic analysis, findings from our interview study show that abortion attitudes are developed through contact with abortion; specifically, experience and/or contact with abortion can make abortion feel more “real” (e.g., tangible, personal, complex, diverse). Recognizing its complexity reaffirmed our participants supportive attitudes towards abortion and highlighted the role that stigma still plays in abortion provision and experiences. This is an under‐researched phenomenon, and we remain hesitant to make claims about the use of contact as a stigma reduction strategy however, given that some research has shown the power of abortion stories to destigmatize abortion and humanize those receiving abortion care, more work in this area will enable us to form clearer conclusions [16]. Our findings underscore the persistence of abortion stigma in United Kingdom and on this basis, we strongly recommend that scholarship continues to explore stigma reduction strategies in the United Kingdom.

## Author Contributions

**Lora Adair:** conceptualization, methodology, formal analysis, investigation, writing – original draft, writing – review and editing, project administration, supervision, funding acquisition. **Julieta Baker:** formal analysis, investigation, writing – original draft, writing – review and editing, writing – revision. **Nicole Lozano:** conceptualization, methodology, validation, formal analysis, resources, data curation, writing – original draft, writing – review and editing co‐supervision. **Aneeka Shrestha:** formal analysis, investigation, data curation. **Ssanyu Kayser:** formal analysis, investigation, data curation.
